# Dynamics of protofibril elongation and association involved in Aβ42 peptide aggregation in Alzheimer’s disease

**DOI:** 10.1186/1471-2105-11-S6-S24

**Published:** 2010-10-07

**Authors:** Preetam Ghosh, Amit Kumar, Bhaswati Datta, Vijayaraghavan Rangachari

**Affiliations:** 1 School of Computing, University of Southern Mississippi, Hattiesburg, MS 39406, USA; 2 Department of Chemistry and Biochemistry, University of Southern Mississippi, Hattiesburg, MS 39406, USA

## Abstract

**Background:**

The aggregates of a protein called, ‘Aβ’ found in brains of Alzheimer’s patients are strongly believed to be the cause for neuronal death and cognitive decline. Among the different forms of Aβ aggregates, smaller aggregates called ‘soluble oligomers’ are increasingly believed to be the primary neurotoxic species responsible for early synaptic dysfunction. Since it is well known that the Aβ aggregation is a nucleation dependant process, it is widely believed that the toxic oligomers are intermediates to fibril formation, or what we call the ‘on-pathway’ products. Modeling of Aβ aggregation has been of intense investigation during the last decade. However, precise understanding of the process, pre-nucleation events in particular, are not yet known. Most of these models are based on curve-fitting and overlook the molecular-level biophysics involved in the aggregation pathway. Hence, such models are not reusable, and fail to predict the system dynamics in the presence of other competing pathways.

**Results:**

In this paper, we present a molecular-level simulation model for understanding the dynamics of the amyloid-β (Aβ) peptide aggregation process involved in Alzheimer’s disease (AD). The proposed chemical kinetic theory based approach is generic and can model most nucleation-dependent protein aggregation systems that cause a variety of neurodegenerative diseases. We discuss the challenges in estimating all the rate constants involved in the aggregation process towards fibril formation and propose a divide and conquer strategy by dissecting the pathway into three biophysically distinct stages: 1) pre-nucleation stage 2) post-nucleation stage and 3) protofibril elongation stage. We next focus on estimating the rate constants involved in the protofibril elongation stages for Aβ42 supported by *in vitro* experimental data. This elongation stage is further characterized by elongation due to oligomer additions and lateral association of protofibrils (*13*) and to properly validate the rate constants involved in these phases we have presented three distinct reaction models. We also present a novel scheme for mapping the fluorescence sensitivity and dynamic light scattering based *in vitro* experimental plots to estimates of concentration variation with time. Finally, we discuss how these rate constants will be incorporated into the overall simulation of the aggregation process to identify the parameters involved in the complete Aβ pathway in a bid to understand its dynamics.

**Conclusions:**

We have presented an instance of the top-down modeling paradigm where the biophysical system is approximated by a set of reactions for each of the stages that have been modeled. In this paper, we have only reported the kinetic rate constants of the fibril elongation stage that were validated by *in vitro* biophysical analyses. The kinetic parameters reported in the paper should be at least accurate upto the first two decimal places of the estimate. We sincerely believe that our top-down models and kinetic parameters will be able to accurately model the biophysical phenomenon of Aβ protein aggregation and identify the nucleation mass and rate constants of all the stages involved in the pathway. Our model is also reusable and will serve as the basis for making computational predictions on the system dynamics with the incorporation of other competing pathways introduced by lipids and fatty acids.

## Background

As in many neurodegenerative diseases, AD is one in which polypeptides aggregate to form amyloid deposits. In AD, the aggregates of a protein called, amyloid-β (Aβ) peptide are strongly believed to be the cause for neuronal death and cognitive decline [1]. Primarily two forms of Aβ, Aβ40 and Aβ42 (40 and 42 amino acids respectively) are observed as the main components of senile plaques in AD patients. Aβ is known to aggregate and form large fibrillar deposits. Among the different forms of Aβ aggregates, smaller aggregates called ‘soluble oligomers’ are increasingly believed to be the primary neurotoxic species responsible for early synaptic dysfunction. 

**Figure 1:**
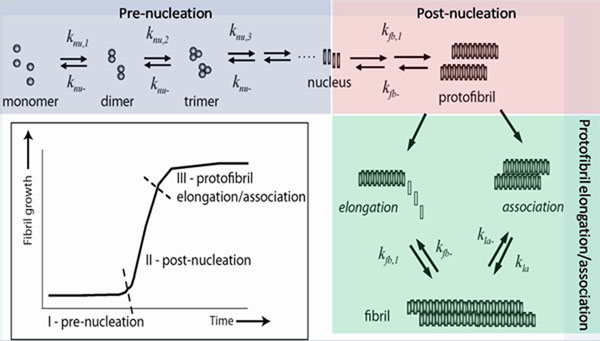
**Kinetics of Aβ aggregation pathway.** Schematic diagram for Aβ aggregation towards fibril formation. (Inset): Typical sigmoidal growth curve of the aggregation process with three largely divided stages. The kinetic parameters associated with stage III (post-protofibril) are examined in this study.

The process of Aβ aggregation is a nucleation-dependent one that was inferred by the occurrence of a ‘lag-phase’ prior to fibril growth showing a sigmoidal pattern [2]. This process involves an initial rate-limiting step of nucleation [3,4] followed by fibril growth [2,5]. A schematic of the process is shown in Figure 1 (Inset). Although the mechanism of nucleation and structure of the nucleus are largely unknown, it has been argued that the structure of the growing fibril is closely dependent on the nature of the nucleus formed. Additional evidence for a nucleation-dependant growth is that the lag-phase can be eliminated by adding pre-formed aggregates to monomers by a process called ‘seeding’ [6]. The efficiency of seeding, which directly reflects the elongation rates, depends on the structure of the ‘seed’ itself [7]. 

Since the structure and shape of the seed is directly linked to the nucleation process, the factors influencing the latter are critical in amyloidogenesis of Aβ. Unfortunately, precise *in vitro* biophysical analyses of Aβ aggregation kinetics are very difficult to achieve. Along the fibril formation pathway, one important intermediate, called protofibrils were identified and characterized [4,8-12] earlier. Protofibrils mainly differ from fibrils in their size and solubility. While fibrils can be sedimented with relatively smaller forces (19000*g*, 10min), protofibrils require substantially high sedimentation forces and have smaller diameters than the fibrils [13]. These protofibrils have propensities to both *elongate* (by monomer addition) as well as to *laterally associate* (protofibril-protofibril association) to grow into mature fibrils. The rates for such process for Aβ40 were also experimentally determined [13]. A more elaborate analysis by O’Nuallain and co-workers reported the kinetics and thermodynamic parameters on Aβ40 protofibril elongation [14]. Besides these, few others reported on the kinetics involved in Aβ aggregation [5,9,15,16]. Nevertheless, there are almost no reports on the biochemical analysis on the pre-nucleation states that reiterates the difficulty involved in such analyses. Also, the kinetics reported in these works report the *aggregate growth rate* of protofibrils by curve fitting methodology, and do not appropriately characterize the rate constants of the biochemical reactions involved in these pathways. Thus, these models and kinetic parameters cannot be re-used to understand the interactions of the protofibrils with the pre-nucleation stage oligomers (monomers, dimmers etc.) and hence cannot give us an exact quantification of nucleation.

One way of overcoming this difficulty is to make theoretical calculations and predictions that can be at least partially validated by experimental data. Based on this principle, Lomakin and co-workers developed a detailed model for determining the rates of nucleation and elongation processes that was supported by light scattering experiments [5,17]. In this model, the authors propose the existence of ‘micellar’ forms of Aβ that is in fast equilibrium with the monomers. Despite these efforts a thorough modeling of Aβ aggregation via molecular-level simulation mechanisms has been lacking. Ideally, any model on Aβ aggregation should be able to identify the nucleation mass and the kinetic rate constants in the different phases of aggregation: pre-nucleation, post-nucleation and fibril elongation. In [25] (and the references therein) the authors provide a detailed review on various models of Aβ aggregation which can be primarily classified as curve fitted models. However, none of these reports present a precise understanding of the process, pre-nucleation events in particular, and have not been computationally modelled either. As fibril formation is often considered to be a stochastic process with a large variation in nucleation rate among identical macroscopic molecules, molecular-level simulations would be essential. Furthermore, it is not realistic to consider aggregation as an isolated event and there are many different factors that can influence protein aggregation in a physiological environment. Broadly, these include molecules that may ‘interact’ with the protein of interest besides others such as ionic strength, pH, temperature etc stressing on the need for reusable models. Only recently, Chung-Lee and colleagues have generated a detailed molecular-level model of insulin aggregation [18], which accurately tries to understand the biophysics behind protein aggregation systems. However, their model cannot be directly used to understand the dynamics of Aβ aggregation wherein, the nucleation stage is itself unknown (more details on this are provided later). 

In a bid to understand the complete dynamics of Aβ aggregation, we propose a divide-and-conquer strategy in this paper by dissecting the Aβ42 aggregation process into three biophysically distinct stages (Figure 1, inset). We justify the importance of understanding each of these stages separately before we can put together a complete model for the aggregation process. In this paper, we present a detailed model of the third stage in the aggregation process that involves protofibril elongation as well as lateral association to fibrils (Figure 1, inset) and report the dynamics in terms of the kinetic rates associated with this stage. Finally, we also discuss how this model for the fibril elongation and association processes can be used to build a complete simulation of overall Aβ42 aggregation towards fibril formation that can accurately identify the rate constants of the other two stages along with the nucleation mass associated with them.

## Results

### Simulation of Aβ fibril formation: Complexities in existing models and biophysical understanding

Simulation of the kind we are presenting in this paper has not been reported for Aβ system. However, Lee and co-workers have elegantly demonstrated such a modelling scheme for insulin aggregation, a system that is fairly similar to Aβ aggregation [18]. Although in their report the authors have indeed presented data for Aβ40, many details were either absent or incomplete. Therefore, in this section, we will review the insulin aggregation model presented in [18] to point out its limitations and motivate our work. 

#### *Biophysical analysis of Aβ42 aggregation.*

**Figure 2:**
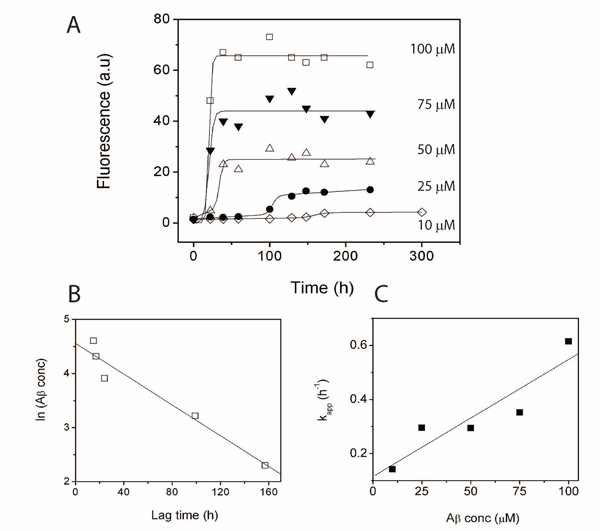
**Characteristics of Aβ aggregation as a function of initial monomer concentration.** Concentration dependence of Aβ aggregation monitored by ThT fluorescence. A) Aggregation in five different initial concentrations of Aβ buffered in 20 mM Tris, 150 mM NaCl, pH 8.0, at 37°C. The data were fit using e*q 1*. B) Relation between the calculated lag-times and initial Aβ concentration and C) relation between rate of elongation and initial Aβ concentration.

First, we monitored the initial concentration dependence of Aβ42 peptides on the aggregation process in five different concentrations; *10, 25, 50, 75 and 100 M* by thioflavin-T (ThT) fluorescence as shown in Figure 2A. ThT is a dye known to exhibit fluorescence upon binding to amyloid aggregates and has become a benchmark for monitoring amyloid aggregation [19]. The objective of this experiment was to estimate lag-times as well as rate of the ‘post-nucleation’ part of aggregation and their dependence on initial Aββconcentrations. To do so, the raw data were fit to equation 1 as previously reported [20], where lag-time is *t_0.5_ – 2b* and the rate constant (*k^app^*) is *1/b*. This rate is assumed to be of the first order and more closely resembles post-nucleation *k_fb_*_,1_ in Figure 1 than pre-nucleation or protofibril elongation stages. Moreover, this rate constant is indicative of the net value for the overall fibril formation and does not represent the individual rate constants for the mult-step reactions in the pathway. The rates and lag-times obtained from fitting the data in Figure 2A with equation 1 are given in Table 1. Since Aβ aggregation is a nucleation-dependant process, increase in its initial concentration will decrease the lag-time and concomitantly increase the apparent rate of aggregation. Thus as expected, we observed the least lag-time and highest apparent rate constants for *100 M* followed by *75*, *50*,* 25* and *10 M* concentrations respectively. In addition, there was an inverse linear correlation between logarithm of Aβ42 concentration and lag-time (Figure 2B). The rate of aggregation was consistent with a first-order kinetics based on the goodness of our fits and a fairly linear dependency of the rate constants on Aβ42 concentration (Figure 2C). These curve-fitted models closely resemble the previous models on the aggregation process and do not study the associated molecular-level biophysics as discussed before.

 (1)

**Table 1:** Lag-time and growth rate constants for Aβ42 aggregation obtained from Figure 2 using equation 1.

Aβ42 (µM)	***k^app^*** (h^-1^)	Lag-time (h)
10	0.14 (± 0.03)	157.34 (± 15.3)
25	0.29 (± 0.15)	99.04 (± 12.1)
50	0.31 (± 0.18)	23.95 (± 7.2)
75	0.35 (± 0.21)	16.61 (± 6.3)
100	0.61 (± 0.31)	15.12 (± 6.2)

#### *Existing simulation on aggregation.*

**Figure 3:**
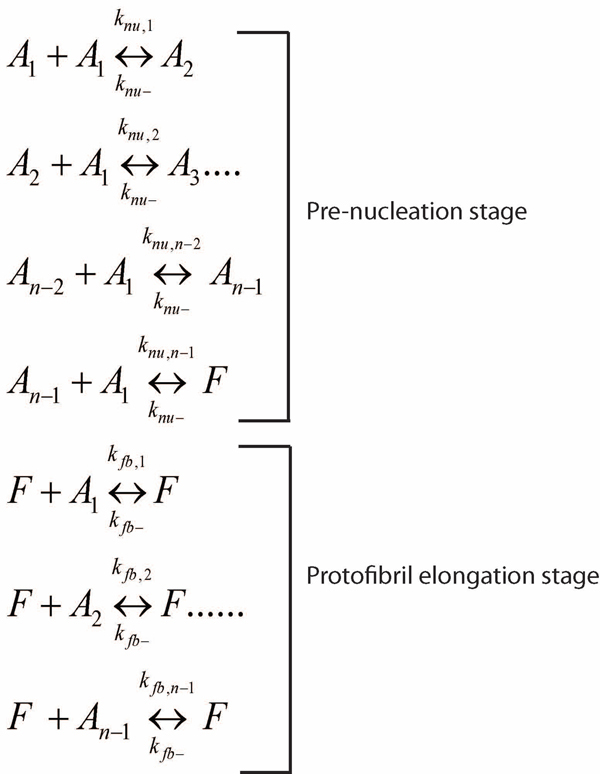
**Reactions towards fibril formation.** Approximate set of reactions to model the entire Aβ aggregation pathway. The reactions are grouped into two stages: a) pre-nucleation stage to model the events occurring before nucleation; these set of reactions are slower and assumed to occur primarily through monomer additions to higher end oligomers due to a higher initial concentration of monomers in the system and b) the protofibril elongation stage consists of a faster set of reactions and is approximated by oligomer additions to protofibrils; we do not consider protofibril-protofibril lateral association reactions in this phase as they are not completely captured by ThT fluorescence.

Molecular level simulations to model the aggregation process typically approximate the pathway by a set of biochemical reactions and compute the corresponding reaction fluxes; next we need to formulate the differential equations for each oligomer concentration as a function of time. This will allow us to study the dynamics (in terms of concentration change) of each of the oligomers involved in the system. The existing model as well as the models proposed in this paper primarily use ODE based molecular level simulations to understand the temporal dynamics and rate constants involved in the pathway. Figure 3 shows the reactions considered along Aβ aggregation towards fibril formation [18]. 

Here, *A_i_*’s denote the *i*-mers, *n* is the nucleation mass and *F* denotes the fibrils. The authors in [18] make the following assumptions to create this model for the insulin aggregation system: *a*) monomer adds to *i*-mers until the formation of fibrils, and *b*) nucleation involves monomer addition as well as a structural change in the oligomer *A_n_*. Post-nucleation events involved during the formation of fibrils are known to be faster than the pre-nucleation stage as inferred from the sigmoidal growth curve (Figure 1, inset). Since nucleation involves a conformational change that serves as a ‘seed’ for further growth, the forward rate constants for post-nucleation is a lot higher than the pre-nucleation ones (i.e., *k_nu,n+i_>>k_nu,i_*). This relative difference in the rate constants can also be attributed to the fact that the larger oligomers produced after the nucleation stage are more likely to interact with the monomers. Lee and co-workers reported a *~10^8^* fold difference in the rate constants for the insulin aggregation pathways [18]. Also, since agitation has been found to drastically shorten the lag-times [23], *k_nu,i_* and *k_fb,i_* are assumed to be diffusion-limited [24]. It should be noted that according to [24], the rate-limiting step is not the conformational conversion but the chance association of a sufficient number of monomers to form a stable, polymerization-competent nucleus. This association of the monomers will be governed by their diffusivities. Following the Stokes-Einstein equation, Lee and co-workers proposed that the diffusivity is roughly proportional to the inverted cubic root of *i*, which equals the size of the cluster and deduced the following:. 

The reverse reaction rate constants are assumed to be independent of size *i*, and are abbreviated as *k_nu-_* and *k_fb_*_-_ respectively. Based on this model, [18] also reported the following estimates of the rate constants for the Aβ aggregation system: 

k_nu,1_= 1.38 ± 0.53 × 10^0^ (h^-1^mM^-1^), 

k_fb,1_= 1.37 ± 1.22 × 10^4^ (h^-1^mM^-1^), 

k_nu-_= 1.01 × 10^-3^ (h^-1^), 

k_fb-_= 3.02 ± 2.64 × 10^2^ (h^-1^).

The primary problem with this model was that it was built for insulin in which the nucleation stage has been well characterized to be less than *n ≤ 10*. Furthermore, Lee and co-workers claimed the nucleation stage of insulin as *6*mers based on their comparisons with the concentration curves of insulin aggregation as it minimized the sum of squared errors (although the results for this was not shown). However, the nucleation state for Aβ aggregation is not known precisely. It is expected that the nucleation stage should be different for different proteins that aggregate. Also, this model approximates the post-nucleation stage by a simplified set of equations without considering the two well-known (and possibly different biophysically) mechanisms of fibril elongation: through oligomer addition and lateral association between protofibrils. Considering, the nucleation mass of Aβ aggregation to be *6*mers, and using the rate constants reported by Lee and co-workers as mentioned above, we get the following comparisons (Table 2) in the lag times from the simulated model and what we have reported experimentally in Table 1. 

**Table 2:** Lag-time comparison between our Aβ42 aggregation simulation and those generated using the model reported in (*18*).

Aβ42 (μM)	Lag-times from our experiments (h)	Lag-times from model in (*18*) (h)
10	158	12
25	99	7
50	24	4.5
75	17	3.5
100	15	2.5

As we find a large difference between the experimentally observed and simulated lag times, we can infer that for the Aβ system, the nucleation mass and rate constants might be quite different. Note that the mapping constant to compare the concentration with the ThT fluorescence intensities from experiments will not have an effect on lag-time estimates and hence cannot explain this difference. Lee and co-workers argue that to quantify the concentration results better, nonlinear effects from at least two possible sources must be considered: the non-ideal behavior of proteins at high concentrations, and a possible experimental artifact from the fluorescence ThT assay [18]. The activity coefficients of proteins at high concentrations are typically not constants and should be considered in the model [21]. Secondly, as mentioned above, nonlinearity with the ThT signal exists perhaps because ThT measurements depend on the ThT:fibril formation, which involves stoichiometric binding of both compounds [22]. This nonlinear relationship is unknown so the proportionality constants based on each curve were assumed to be different. This however is not a reasonable argument as we discuss later.

### Simulation complexity of the on-pathway

Thus in order to use the same model for the Aβ system, we need to estimate the following six unknown variables: *k_nu,1_, k_fb,1_, k_nu-_, k_fb-_ , n* and *b*, where, *b* is the constant that maps ThT fluorescence to concentration estimates. It is certainly difficult to iterate through different values for each of these variables to get close to the experimental plots (as done in [18]) due to the huge solution space and finding the nucleation phase (i.e., *n*) cannot be done independently without having estimated the rate constants and mapping constant alongside. This important part of the problem has not been discussed sufficiently in [18] either. 

Our approach in this paper is to employ a divide-and-conquer strategy to bring down the number of variables to be estimated together and thereby reduce the search space as we discuss next. We first dissect the sigmoidal fibril-growth curve into the following three sections based on both experimental viability and the ease of modeling (schematic shown in Figure 1 inset): (i) pre-nucleation stage (ii) post-nucleation stage and (iii) protofibril elongation stage. The pre- and post-nucleation stages can be well-approximated by the set of reactions shown in Figure 3. However, the protofibril elongation stage needs to combine both the post-nucleation and lateral association stage reactions [13] that were not considered in Lee *et al*’s model. However, considering the lateral association mechanism actually *increases* the complexity of the model as we now have to estimate two more parameters in addition to the previous six: the forward and backward rate constants for the lateral association stage denoted by *k_la_* and *k_la-_* respectively. 

Our goal is to estimate the post-nucleation rate constants (*k_fb,1_, k_fb-_, k_la_* and *k_la_*) separately that can be verified by fibril seeding experiments without having to consider the pre-nucleation stage variables. In this paper, we report our estimates for these four rate constants that were accomplished by building a different reaction model, validated by *in vitro* data. This work formulates a fundamental basis for our ultimate goal of estimating all the kinetic parameters along the aggregation pathway towards fibril formation and identifying the nucleation mass(es) for Aβ aggregation.

### Modeling the protofibril elongation stage

#### Biochemical analyses of Aβ42 protofibril elongation

**Figure 4:**
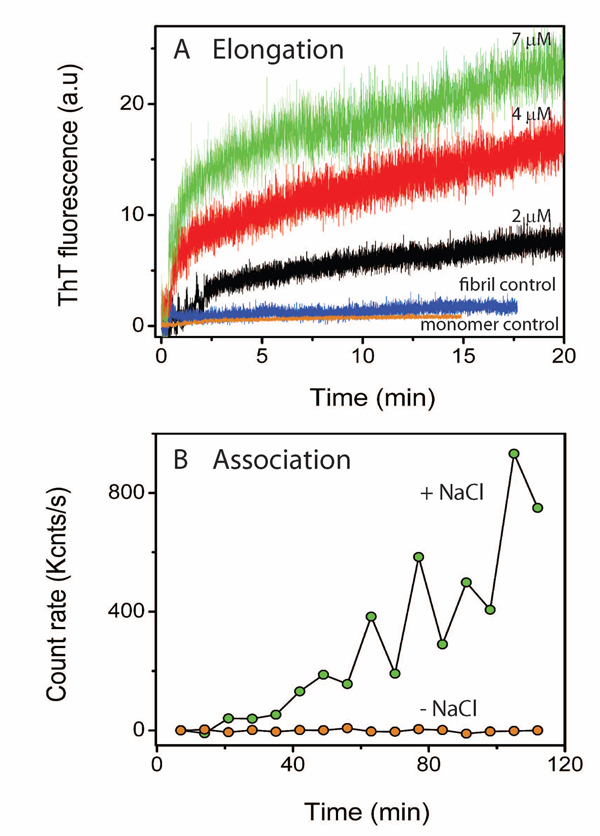
**System dynamics from the biochemical experiments conducted for protofibril elongation and lateral association.** Experimental results for Aβ42 protofibril elongation and lateral association. A) Three concentrations of isolated protofibrils (7, 4 and 2 µM) were elongated with freshly purified Aβ42 monomers (30 µM). The reactions were monitored by ThT fluorescence. Both mature fibrils and monomers (~ 4 µM) were used as negative controls. B) Lateral association was initiated by the addition of 150 mM NaCl to the isolated protofibrils (4 µM) that was monitored by Dynamic Light Scattering instrument. Protofibrils without salt was used as a negative control. The data is plotted against count rate which is directly proportional to the hydrodynamic radius of the sample.

Synthetic Aβ42 peptide was obtained from synthesis facility at Mayo Clinic, Rochester, MN as a lyophilized powder. Aβ42 protofibrils were generated and isolated as previously reported [11]. Freshly purified Aβ42 monomers (*100 µM*) from size exclusion chromatography buffered in *10 mM* Tris, *50 mM* NaCl, pH *8.0* was agitated at room temperature for *48 h*. The aggregation was monitored using ThT fluorescence. The sample was then centrifuged at *19000g* for *12 min* to spin out any fibril that may have formed. The supernatant was then fractionated by Superdex-*75* size exclusion column to isolate protofibrils from unreacted monomers and smaller oligomers. The concentration of protofibrils was measured by UV-Vis with a molar extinction coefficient of *1450 cm^-1^ M^-1^* corresponding to Aβ42. The elongation reactions were initiated by adding *2, 4* or *7 µM* protofibrils to *30 µM* freshly purified, seed-free monomers and with *10 mM* ThT, in a fluorescence cuvette (total volume of *100 µl*) (Figure 4A). The initiation of the elongation reaction is carried out under low salt conditions (*50 mM*) to minimize any protofibril-protofibril association. Hence, the addition of monomers to protofibrils under these conditions will lead to an increase in the size of protofibrils that can be monitoted by ThT fluorescence. The results from this phase are shown in Figure 4A. It should be noted that ThT will exclusively give response to elongation reactions and will not detect association reactions. This is because, ThT is known to bind between two monomer units and association reactions do lead to the formation of new monomer-protofibril units, as opposed to the elongation reactions.

### ODE-based molecular level simulation of protofibril elongation phase

**Figure 5:**
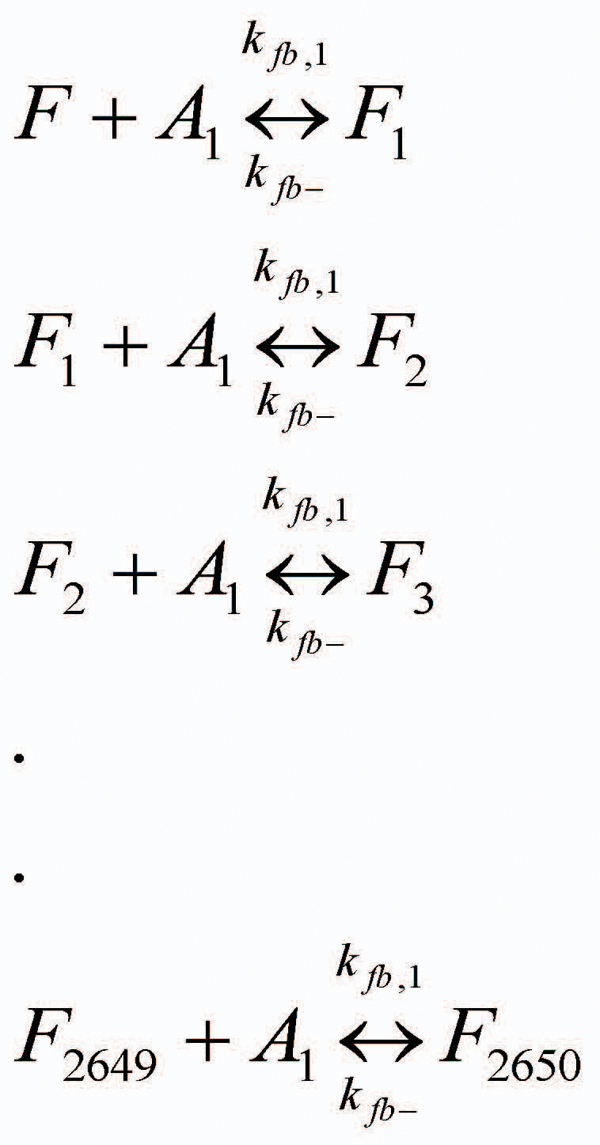
**Protofibril elongation reactions - oligomer addition phase.** The oligomer addition phase in protofibril elongation is approximated by monomer additions only. Due to the high initial monomer and protofibril concentration in the system, these set of reactions will primarily govern the system dynamics during the early phases (~0-10 mins) of protofibril elongation.

**Figure 6:**
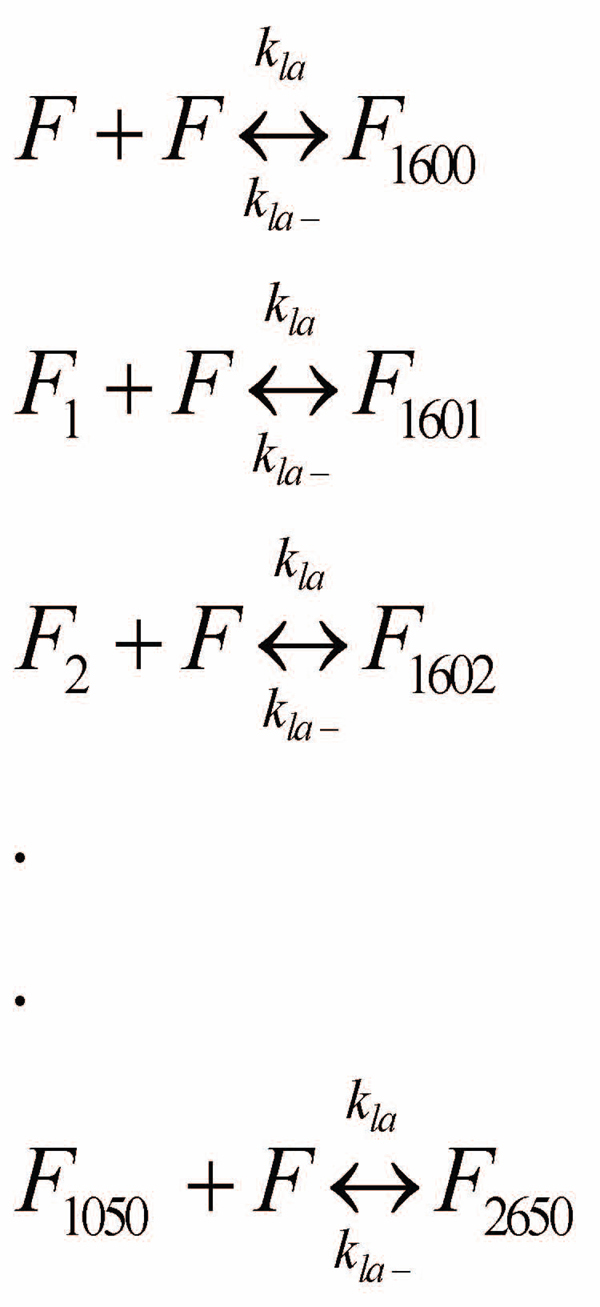
**Protofibril elongation reactions – protofibril-protofibril lateral association phase.** The lateral association phase is modelled by protofibril association to the elongated protofibrils from the oligomer addition phase. We do not consider variable length protofibril association in this phase to reduce the system complexity. These set of reactions can model the lateral association stage accurately in the initial phases (~0-10 mins) with high concentration of standard protofibrils (i.e., *F*).

The data thus obtained was modeled using Matlab’s ODE toolbox. In particular, we have considered the set of reactions shown in Figures 5 and 6. Here, *A_1_* denotes an Aβ monomer and *F* are the protofibrils (of length *~1600-*mers and average length *~64 nm)*[11]. Also, *F_i_* denotes a protofibril with *i* number of Aβ molecules binding to it during the elongation phase. 

Nichols and co-workers showed that typically the elongation stage saturates with the formation of *F_i_*’s with length in the range *160-180 nm*. Considering the average *F_i_* length, at saturation, to be equal to *170 nm*, we can compute the number of Aβ’s attaching to a protofibril in the elongation phase as: *(1600x170/64 – 1600) = 2650*. Furthermore, *F* denotes a standard protofibril of length *~1600-*mers, whereas the *F_i_*’s denote elongated protofibrils of length *~(1600+i)*-mers.

Figure 5 shows the oligomer addition reactions which are similar to the post-nucleation phase reactions considered in the model shown in Figure 3. However, we only consider monomer addition in this phase so that we can eliminate the nucleation mass estimates and keep the number of parameters to be modeled simple. This is certainly an approximation of the over-all oligomer addition characteristics of the fibril elongation phase, yet it is sufficient to appropriately estimate *k_fb,1_* because of the following: 

• The seeding experiment was intentionally done over a shorter time period of *30-60 m*.

• It is safe to assume that with *30 μM* initial concentration of Aβ monomers, we will not have sufficient number of nucleated oligomers in the system. The pre-nucleation stage rate constants being a lot slower than the post-nucleation ones and considering a sufficiently high initial monomer concentration (of *30 μM*), the number of dimeres, trimers and other oligomers formed in the system will be relatively low leading to lesser number of pre-nucleation reactions.

• Considering the protofibril growth curve over a shorter time span (we have considered protofibril growth for *1 min, 2 mins* and *5 mins* to estimate the rate constants), we can further eliminate the effects of these lower-end oligomers in the system.

• The model assumes homogeneous distribution of the oligomers and protofibrils in the system (that can be simulated by constant stirring) to alleviate the needs of considering the spatial effects and molecular crowding phenomenon.

Hence, essentially we further dissect the fibril elongation growth curve due to seeding so as to eliminate the effects of the other unknown parameters in the system. This methodology can give us an estimate of *k_fb,1_* and *k_fb-_* without requiring us to estimate the other rate constants and nucleation masses in the pathway. The complete set of results for *30-60 mins* will then be used to estimate the lateral association stage rate constants only, as we would have already estimated the post-nucleation stage rate constants by then.

For each reaction shown in Figure 5, we have *k_fb,1_* denoting the rate constant for the reaction leading to the formation of *F_i_*. Note that, in contrast to the post-nucleation reactions shown in Figure 3, we do not have to use the Stokes-Einstein equation to relate the forward rate constants in the elongation stage. This is because our assumption of monomer-additions to protofibrils towards the beginning of the fibril elongation phase does not require us to consider the effects of other oligomers in the system. Also, the protofibril sizes being relatively a lot larger than that of individual monomers, the effects of protofibrils (of different sizes) on the diffusivity of the reactants will be quite similar. Indeed the Stokes-Einstein equation relating the rate constants in the post-nucleation phase were related as follows (from [18]): , where the rate constants only differed due to the sizes of the smaller oligomers in the system, which in our case are of the same size (i.e., *i=1*). The rate constants for the reverse reactions are assumed to be independent of size *i*, and abbreviated as *k_fb-_* following the discussion in [18]. Thus, we have *three* unknown variables (*k_fb,1_, k_fb-_* and the mapping constant, *b*) from the reaction set in Figure 5 that we need to estimate by fitting to the experimental data. 

**Figure 7:**
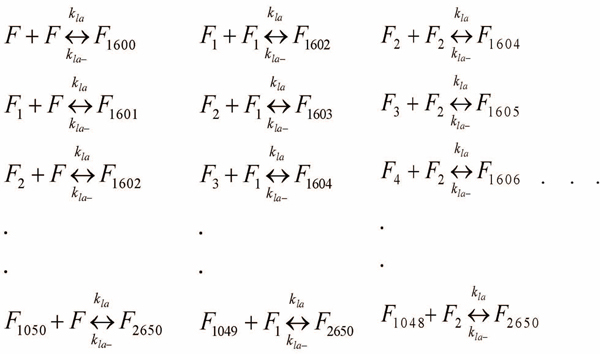
**All possible lateral association reactions.** This figure shows all possible lateral association reactions resulting from variable sized protofibrils associating amongst themselves. These reactions illustrate the complexity of the lateral association phase that can potentially result in an infinite set of reaction combinations.

The reactions shown in Figure 6 model the lateral association stages. Ideally, lateral association should involve the association of all possible sizes of protofibrils to each other as illustrated in Figure 7. However, this will exponentially increase the number of reactions that need to be simulated and hence the simulation complexity. To address this issue, we will model the lateral association stage by the reduced set of reactions of Figure 6, where we restrict ourselves to only the standard protofibrils (of length *~1600-mers*) to react with the larger ones formed due to monomer additions from Figure 5. This assumption will again be valid towards the beginning of the elongation phase (initial *30-60 mins*) wherein the number of standard protofibrils (i.e., *F*) and monomers significantly outnumber the intermediate protofibrils (i.e., *F_i_*’s) and other oligomers (*A_i_*, *i*=*2,…,n*) in the system. 

The actual reaction fluxes for each of the above mentioned models along with the corresponding differential equations are reported in the Methods section.

### Simulation results

In this section, we report the performance of our models and our estimates of the 5 unknown parameters for the fibril elongation phase. All the simulations were executed in Matlab’s ODE solver (we have used ode23s). Our methodology involves iterating each of the forward rate constants from *10^-6^,…10^8^* and the backward rate constants from *10^6^,…10^-8^* at multiples of *10* (a total of *15x15=225* test cases for a particular estimate of *b*) to identify the best combination that gives us the least square error in comparison to the experimental estimates. Before discussing the results, let us first explain the mapping of ThT fluorescence intensities to the concentration curves of different species in the elongation phase.

### Comparing concentration curves to ThT fluorescence intensities

The fluorescence intensity plots from experiments essentially show the *cumulative effect* of all protofibrils of a certain size (and beyond). It is well known that protofibril elongation is accompanied by an increase in ThT fluorescence while lateral association does not [13]. This is because as lateral association involves attachment of two protofibrils (Figure 1), it does not result in the generation of new ThT binding sites, contrary to the elongation mechanism. Therefore, we can safely assume that a majority of ThT signals observed in elongation experiments do not reflect any lateral association process. Hence, from the simulation, one has to plot the cumulative effects from all the protofibrils that can be mapped directly to the experimental estimates. In order to do this we will compute the following expression at each value of the simulation time:

where *[F_i_]* denotes the concentration of *F_i_*, *i* denotes the number of Aβ molecules by which the fibril has elongated and hence the contribution of the corresponding *F_i_* towards the fluorescence, and *b* is a constant scaling factor to map to the fluorescence sensitivity estimates. 

Note that we have not considered the concentration of the initial protofibrils (i.e., *F*) in the above expression and the cumulative estimate is recorded only for the *elongated* protofibrils. The major reason behind this is the fact that although the average size of the protofibrils is assumed to be *~1600-mers*, it is only an average estimate and hence it might not be correct to bring in its effects by multiplying *[F]* by *1600*. In fact, some of the initial fibrils used in the experiments might already have elongated (or laterally associated) even before the experiments were conducted. To get around this problem, we have preprocessed the experimental data by subtracting each fluorescence intensity (for each time point) for every concentration by the minimum intensity value for that specific initial concentration of protofibrils. This scales down the intensities to start from zero and eradicates the effects of the initial protofibril concentration on the fluorescence intensity. Thus, the intensity values as well as the cumulative concentration (shown in the results) start from zero in the y-axis. 

Also, as the experimental set-up (and hence the conditions) for generating the intensities against different initial concentrations was the same, it is unlikely that the mapping constant *b* should be different for different initial concentrations. This is in contrast to what has been proposed in [18] to fit the curves conveniently to experiments without considering the intricacies of the system of equations. Our results have been generated keeping the value of *b* constant and set to *b=2000* that gave the least square error after several additional iterations of the Simple Model. 

### Simulation results and rate constant estimates

#### Estimates for monomer addition phase

**Figure 8:**
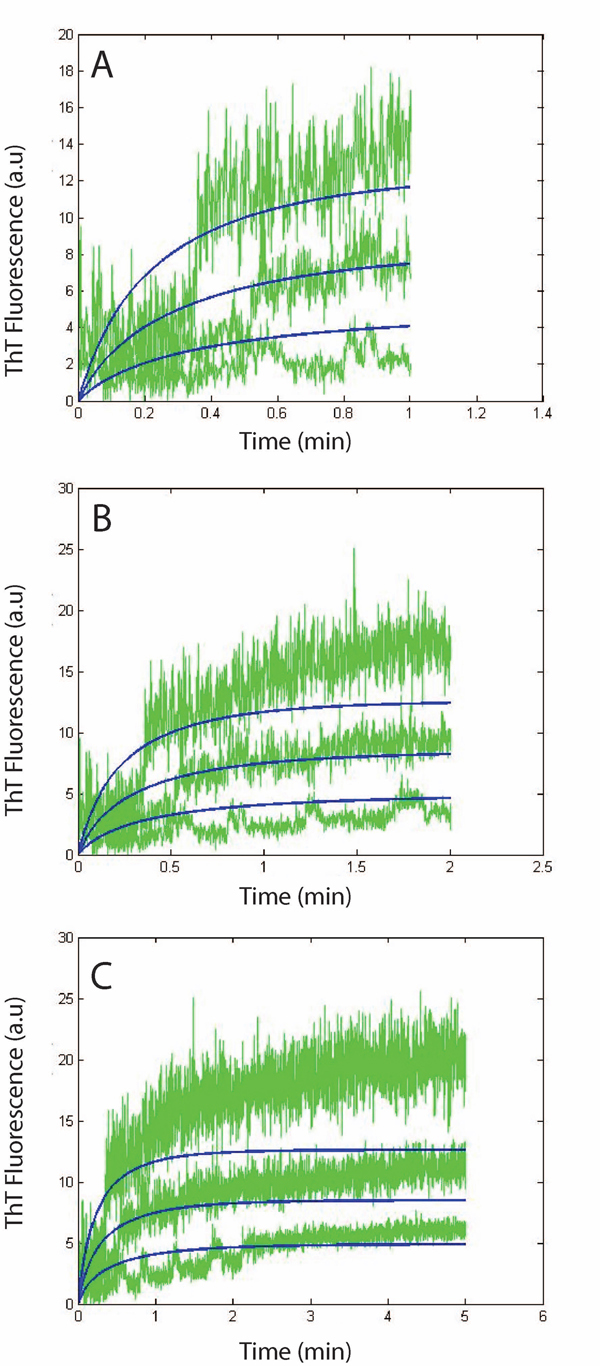
**System dynamics for 1-5 mins: Comparison between simulation results using the simple model and experimental plots generated by ThT fluorescence.** Simulation vs experimental data for protofibril elongation over 1 min (A), 2 min (B) and 5 min (C). Green curves are the elongation data for isolated protofibrils (2 µM, lower curve; 4 µM, middle curve; 7 µM, upper curve) elongated by freshly purified Aβ monomers (30 µM). Simulation plot modeling the elongation phase using simple model (blue).

We iteratively simulated the Simple Model first to estimate the rate constants for the monomer addition phase. To compare with the experimental results, we only considered the fluorescence curves upto *1 min* (Figure 8A), *2 mins* (Figure 8B) and *5 mins* (Figure 8C) as the effects from lateral association will be higher after that. The best fit to the experimental plots (measured in terms of least squared error) yielded the following estimates: *k_fb,1_=9.0x10^3^ h^-1^mM^-1^, k_fb-_=4.5 x10^2^ h^-1^* and *b=2000* respectively. 

Figure 8A shows very good correspondence to experimental estimates as expected (*R^2^=0.99*), as the lateral association or higher-end oligomer formation effects are minimal at the early phases of the elongation stage. However, as the system is allowed to evolve for a larger amount of time, the Simple Model becomes increasingly less accurate (*R^2^=0.86* for *2 mins* and *R^2^=0.7* for *5 mins*) due to these effects (as seen in Figures 8B and 8C). The *R^2^* was computed by averaging out the *R^2^* estimates for each of the *3* concentrations considered for protofibrils (i.e., *2 μM, 4 μM* and *7 μM*) for a constant *30 μM* of initial monomer concentrations.

Figure 8C points to another important aspect of the system dynamics. We find that with lower initial protofibril concentration, the effects of higher system time (*~5 mins*) are not as much as with higher concentrations. This would logically mean that the results from the Simple Model worsen with higher system time because of primarily the lateral association reactions (and not higher-end oligomer addition to protofibrils) Note that, with higher fibril concentration, the rate of monomer consumption will be correspondingly higher and one can argue that this will prohibit higher end oligomers to be formed quickly in the system. However, the initial monomer concentration being relatively quite higher than that of the protofibrils, we should still have enough monomers that escape the elongation phase and start the oligomer formation process. This corroborates our assumption in the Simple Model that the higher-end oligomers do not have a pronounced effect in the fibril elongation dynamics within the first few minutes.

### Effects of the mapping constant b

As mentioned before all the simulation plots were generated keeping *b* constant (*=2000*) as we discussed the importance of this parameter. If we vary *b* to better fit the experimental plots for different estimates of initial protofibril concentrations following the methodology in [20], we do get a better *R^2^* estimate; however, that might not be practical.

**Figure 9:**
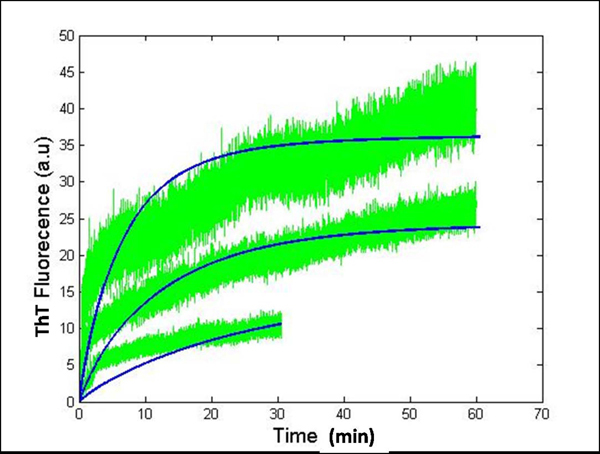
**System dynamics for 60 mins using simple model and varying mapping constant, *b*.** Simulation vs experimental data for protofibril elongation for a longer time-span. Isolated protofibrils were elongated using freshly purified Aβ monomers (green). Simulation plots modeling the elongation phase for longer time-scales (blue) for k_fb_= 1.08x10^3^ h^-1^mM^-1^ and k_fb-_= 1.2 x10^1^ h^-1^. These plots were generated by varying the mapping constant *b* for different initial concentration of protofibrils as follows: *b=800* for *2* µM of F, *b=1200* for *4* µM of F and *b=1700* for *7* µM of *F*.

Figure 9 shows the fit of the Simple model to the experimental plots and we find a relatively high *R^2^=0.99*. To generate the plot we have used the following estimates for the rate constants and *b*:

k_fb_= 1.08x10^3^ h^-1^mM^-1^; 

k_fb-_= 1.2 x10^1^ h^-1^;

b = 800 (for 2 μM initial protofibril concentration)

b = 1200 (for 4 μM initial protofibril concentration)

b = 1700 (for 7 μM initial protofibril concentration)

The initial monomer concentration was always fixed to *30 μM* as discussed before. The apparent non-linearity in the mapping of concentration estimates to ThT fluorescence (as discussed in [18]) seems to increase with higher protofibril concentration. However, keeping *b* constant at *2000*, gave us the best fit for all the *3* protofibril concentrations used pointing to the fact, that this non-linearity might not be directly proportional to the initial concentrations and might be a vague assumption. 

 Also, Figure 9 was generated using the Simple model and we know that the set of reactions considered in Figure 5 cannot accurately explain the dynamics of the elongation stage for sure (because of the contribution from the higher-end oligomers in the system and lateral associations). Thus the mapping constant *b* is actually a very important parameter that allowed us to *fit* our Simple model to the experimental plots (and get good agreement with it) without precisely modeling all possible reactions in the pathway. Hence, until a physical basis for varying the mapping constant is established, we believe it to be prudent to use a constant *b* for any subsequent work that requires a correspondence of concentration estimates from simulations to ThT fluorescence curves from experiments.

### Estimates for lateral association phase from ThT fluorescence experiments

To estimate the rate constants for the lateral association phase, we simulated the Complete Model setting *k_fb,1_=9.0x10^3^ h^-1^mM^-1^, k_fb-_=4.5 x10^2^ h^-1^* and *b=2000* as approximated above and iterating through different values for *k_la_* and *k_la-_* (*225* test cases) as mentioned before.

**Figure 10:**
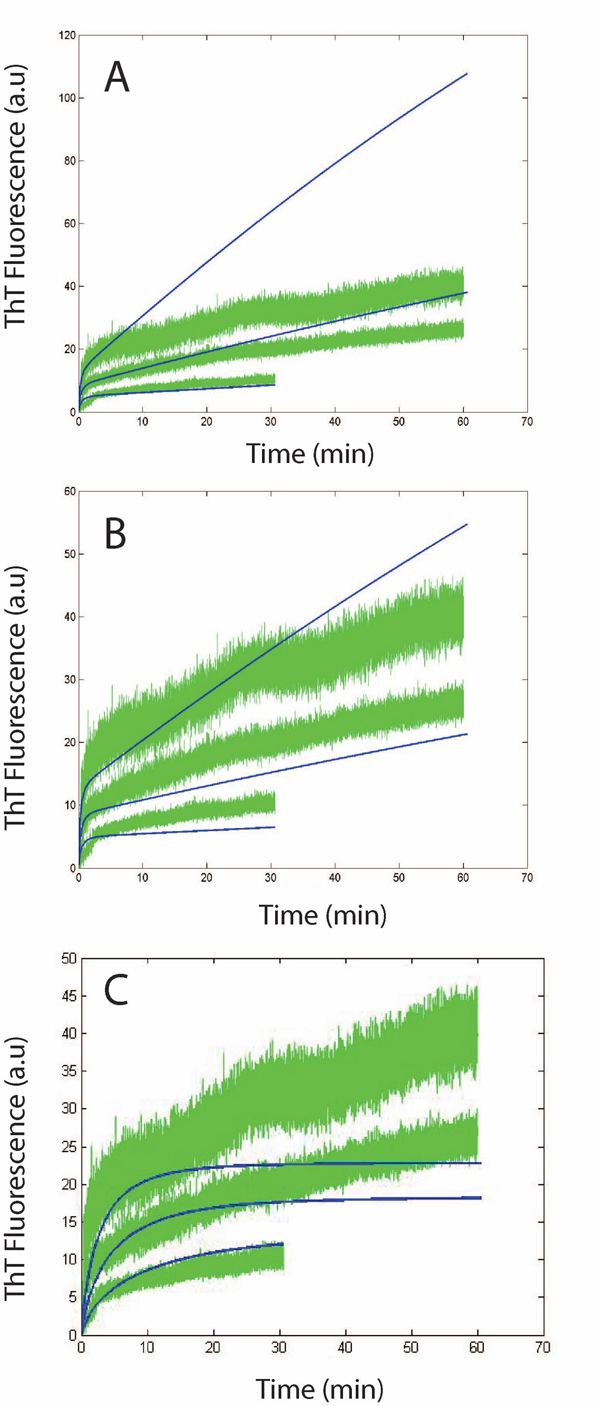
**System dynamics for 60 mins: Comparison between simulation results using the complete and simple models and experimental plots generated by ThT fluorescence.** Simulation vs experimental data for protofibril elongation from the Complete model. (A) results generated for k_la_= 2.1 h^-1^mM^-1^ and k_la-_= 6.0 x10^-3^ h^-1^. (B) k_la_= 9.0x10^-1^ h^-1^mM^-1^ and k_la-_= 6.0 x10^-3^ h^-1^. (C) simulation results from the Simple model for longer time-span. Isolated protofibrils were elongated using freshly purified Aβ monomers (green). Simulation plots modeling the elongation phase for longer time-scales (blue).

Figures 10A and 10B shows the system dynamics with the Complex Model and rate constant estimates of *k_la_= 2.1 h^-1^mM^-1^* and *k_la-_= 6.0 x10^-3^ h^-1^, R^2^= 0.68* and *k_la_= 9.0x10^-1^ h^-1^mM^-1^* and *k_la-_= 6.0 x10^-3^ h^-1^, R^2^= 0.6* respectively. The low *R^2^* estimates point to the importance of considering all possible lateral association reactions as we have showed in Figure 7 at the cost of very high computational complexity. One important distinction of our models with the ones proposed in [18] is that we are estimating the rate constants based on multiple curves due to different initial concentrations which will allow us to get closer to the actual rate constants involved in fibril elongation. Alongside, we always keep *b* constant as discussed above which is more practical. The model in [18] gives higher *R^2^* estimates only for a particular initial concentration and fail for other cases keeping *b* constant (as we have shown in Table 2).

 We believe that the rate constants corresponding to Figure 10B might be a better estimate (although it gives a lower *R^2^* value) of the lateral association rate constants due to the nature of the curves. The model becomes less accurate as the system time increases due to the other lateral association reactions that we have not considered and also due to oligomer addition to protofibrils effects. These additional reactions will produce larger protofibrils quickly and could have pushed up the simulation curves closer to that of the experiments. Also, as before, the worsening is less for lower initial concentrations of protofibrils as that will have a reduced effect on the lateral association reactions. 

**Figure 11:**
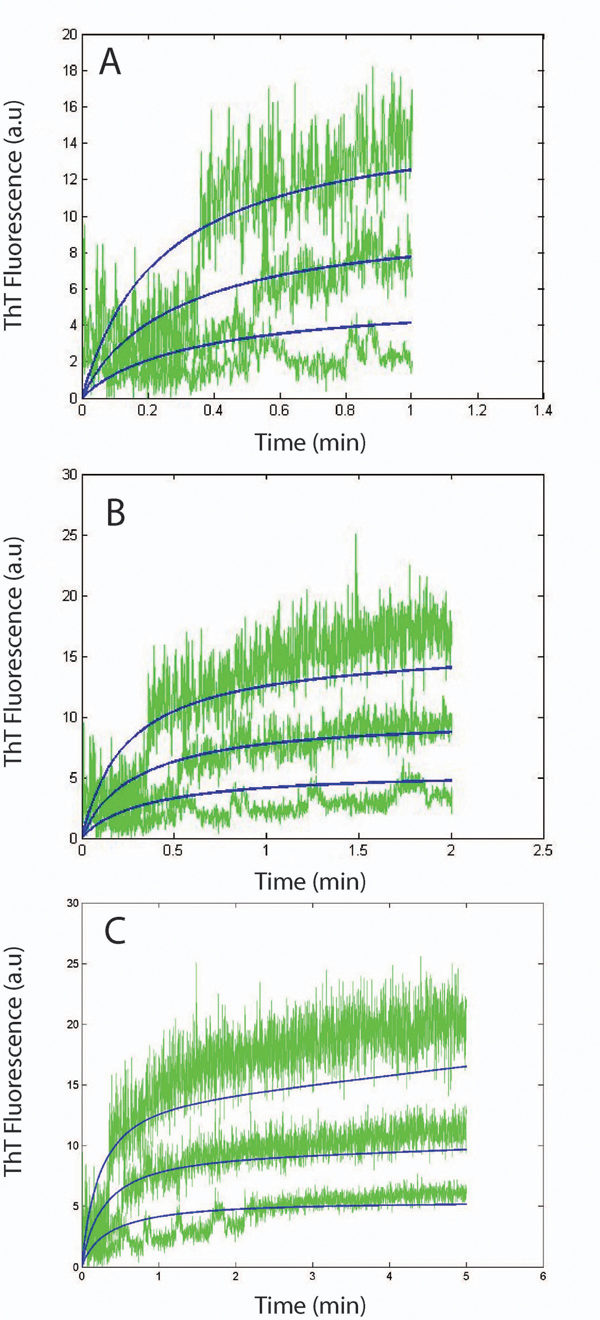
**System dynamics for 1-5 mins: Comparison between simulation results using the complete model and experimental plots generated by ThT fluorescence.** Simulation vs experimental data for protofibril elongation over 1 min (A), 2 min (B) and 5 min (C). Isolated protofibrils were elongated as mentioned in Figure 8. Simulation plot modeling the elongation phase using complete model (blue).

Another interesting observation is that for a shorter time span (*1 min, 2 mins* and *5 mins*), the Complete model performs quite well (based on the rate constants estimated from Figure 10B) showing the validity of our estimates (as can be seen in Figures 11A, 11B and 11C). In particular, the *R^2^* for a *1 min* run of the system is *0.99* and only goes down to *0.95* and *0.87* for *2 mins* and *5 mins* respectively. However, for longer time-spans, the results from the Complete model worsen appreciably. Specifically, as seen in Figures 10A and 10B, the Complete model tends to overestimate the protofibril effects on the ThT fluorescence. This is because of the fact that the experimentally generated ThT fluorescence intensity generally fails to capture the contributions from the lateral association phase as discussed before. Thus we need to validate our kinetic estimates for this phase using Dynamic Light Scattering (DLS) experiments as mentioned next. 

### Estimation of Aβ42 protofibril-protofibril association kinetics by DLS

**Figure 12:**
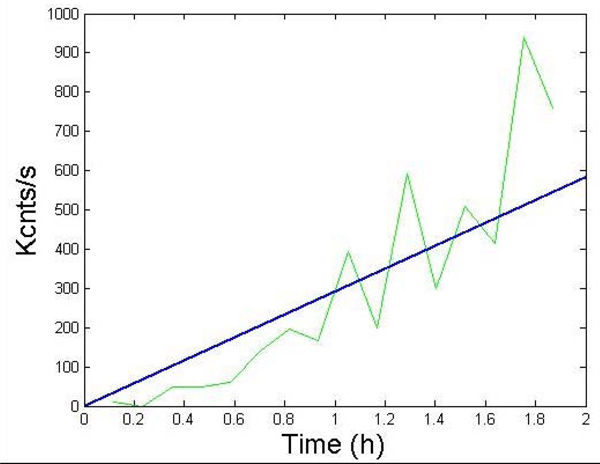
**System dynamics from the stand-alone lateral association model presented in Figure**13. Simulation vs experimental data for protofibril-protofibril lateral association reactions for 2 hrs. Isolated protofibrils were laterally associated and their count rate (green) measured by DLS. Simulation plots modeling the association phase (blue) for k_la_= 9.0x10^-1^ h^-1^mM^-1^ and k_la-_= 6.0 x10^-3^ h^-1^.

Aβ42 protofibrils were generated as mentioned before. The reactions were performed on size-exclusion chromatography-purified protofibrils. The lateral association reaction was initiated by addition *150 mM* NaCl on *4 μM* Aβ42 protofibrils in *10 mM* Tris, pH *8.0* in a total volume of *70 μl* and was monitored for* 120 min* on a Zetasizer nano S DLS instrument (Malvern Inc., Worcestershire, UK). The average count rate (Kcnts/s) was plotted against time to generate Figure 12 (green). Elongation of appropriate control without salt was also performed that is indicated in the same figure indicating no association. The results from these DLS experiments are shown in Figure 4B. 

### A separate model for protofibril-protofibril lateral association that can be validated by DLS experiments

**Figure 13:**
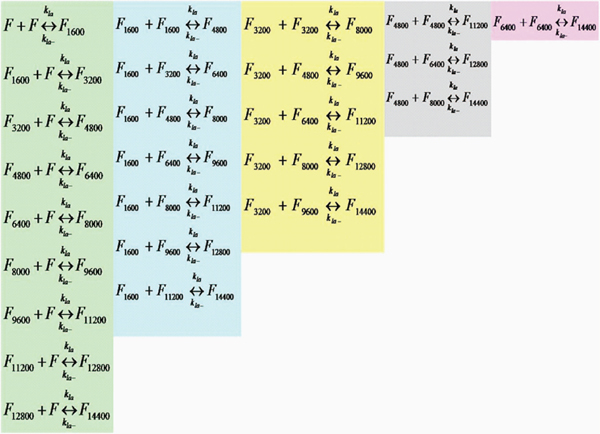
**All possible lateral association reactions considering at most 10-fold increase in the size of protofibrils.** This figure shows all possible lateral association reactions considering at most a 10-fold increase in the original protofibril size. Also, these set of reactions model the lateral association phase explicitly without any oligomer addition taking place.

In this section, we present a separate model targeted towards the lateral association phase only. Note that as mentioned in the DLS experimental set-up, this phase does not involve monomers (and hence any protofibril elongation reactions). Figure 13 shows the reactions involved under the assumption of a homogeneous mixture of 4 µM protofibrils used in the *in vitro* experiments. The *10* different molecular species involved in this phase are *F*, *F_1600_*, *F_3200_*, *F_4800_*, *F_6400_*, *F_8000_*, *F_9600_*, *F_11200_*, *F_12800_*, and *F_14400_*. We assume that lateral association will result in at most a 10-fold increase in size of the protofibrils within the first 2 hrs of observation such that the system of reactions will not involve the formation of protofibrils beyond *F_14400_*. This is a rough estimate though as we find approximately a 5-fold increase in *Kcnts/s* measurements from DLS.

**Figure 14:**
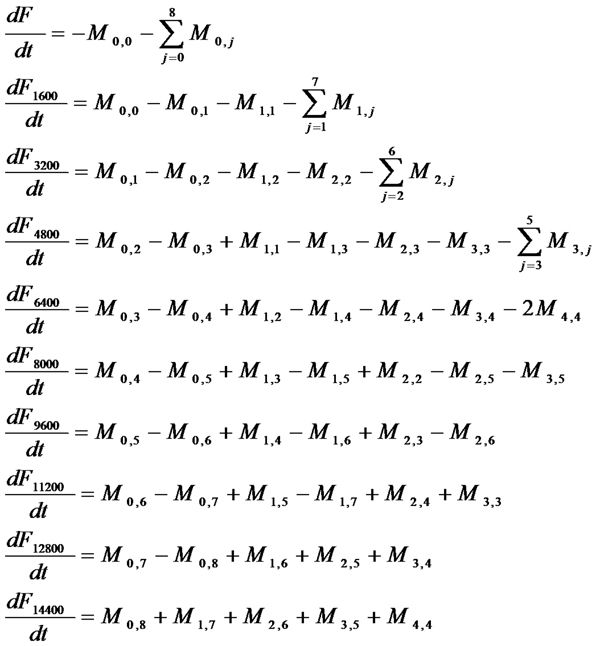
**Differential equations for the lateral association reactions considering at most 10-fold increase in size of protofibrils and no fibril elongation reactions involved.** This figure shows the differential equations governing the concentration change of the protofibrils corresponding to the reactions shown in Figure 13. We assume a maximum 10-fold increase in size in the protofibrils and no oligomer addition taking place in the system due to the absence of monomers.

The reaction fluxes in this phase are denoted by *M_i,j_* corresponding to the reaction between F_i*1600_ and F_j*1600_ forming F_(i+j+1)*1600_ for *i=0,1,…,4* and *j=i,i+1,…,8-i* where for simplicity of notations we denote *F* by *F_0_*. Thus, we get:

The corresponding differential equations for this phase are shown in Figure 14. Note that it is actually hard to generalize these set of reactions and hence we will only be using this model to validate our previously determined estimates of *k_la_* and *k_la-_*. The DLS experimental results were preprocessed as before by subtracting the minimum *Kcnts/s* value from all the data points. Correspondingly, we use the same scheme for mapping the concentration estimates to that of DLS and do not consider the contributions from the initial protofibrils (i.e., *F*) as the summation starts from 1. However, in this case, we only need to sum the weighted concentrations of the *9* distinct molecular entities as shown above that are formed in this system. Figure 12 shows the comparison of the DLS experimental results with the simulation plot for *k_la_= 9.0x10^-1^ h^-1^mM^-1^* and *k_la-_= 6.0 x10^-3^, b=6500* and a relatively high *R^2^=0.89*. Note that we have used a different estimate for the mapping constant b in this case as the DLS experiments are substantially different from that for ThT fluorescence.

The lateral association model (from Fig 13) predicts a linear increase in the count rate (and hence size) of the protofibrils which is quite similar to the experimental observations. An even better illustration of this can be found in [13,26] where the authors have conducted a number of light scattering experiments on Aβ40 (having similar characteristics as Aβ42) and reported such linear growth of protofibril size for ~100 hrs. Also, since protofibril growth also needs to saturate at some point of time we used this model for a longer time-scale and found the growth to saturate after about 3000 hrs (results not shown). Such predictions are however hard to validate experimentally. 

## Discussion

### Contribution of our rate constant estimates to the overall simulation of the Aβ42 aggregation pathway

It is also worth mentioning that over-all the rate constants for the lateral association phase is about *10,000* times slower than that of the monomer addition phase and hence the model proposed in [18] might still be able to simulate the complete dynamics of the Aβ aggregation system even though it does not explicitly consider lateral association. Also, with our estimates of *k_fb,1_* and *k_fb-_* we have reduced the number of unknown parameters and hence the complexity of this model that makes it more plausible to estimate the nucleation mass and the pre-nucleation stage rate constants.

In Figure 10-C, we have used the Simple model to fit the experimentally generated fluorescence curves for a longer time-span. As expected, these results are worse than what we see from the Complete model, and actually points to the importance of considering the lateral association reactions which was not done in [18]. However, our next endeavor of simulating the complete fibril formation pathway (considering pre-, post-nucleation and fibril elongation stages) might get prohibitively complex in terms of computational requirements if we are to consider the complete sets of reactions. Hence, Figure 10-C approximates the complete fibril elongation dynamics using the Simple model (and hence a reduced set of reactions) for which the best fit was obtained for *k_fb,1_=1.5x10^3^ h^-1^mM^-1^, k_fb-_=4.5 x10^1^ h^-1^* and *R^2^=0.58*. These estimates could be used to approximate the fibril elongation phase with a reduced set of reactions while simulating the entire fibril formation pathway.

## Conclusions

In this paper, we have proposed a divide-and-conquer strategy to estimate the fibril elongation dynamics of the Aβ protein aggregation system. In order to understand the kinetics of the entire fibril formation pathway, our strategy dissects the sigmoidal fibril growth curve into 3 stages: pre-nucleation stage, post-nucleation stage and fibril elongation stage. We have presented an instance of the top-down modeling paradigm where the biophysical system is approximated by a set of reactions for each of these stages. In this paper, we have only reported the kinetic rate constants of the fibril elongation that were validated by *in vitro* biophysical analyses. Specifically, the time-line of the *in vitro* experiments on fibril elongation was further subdivided to approximately identify the kinetics of the separate facets of protofibril elongation: oligomer addition stage (approximated by monomer additions in this paper) and lateral association stage. The kinetic parameters reported in the paper should be at least accurate upto the first two decimal places of the estimate. We sincerely believe that our top-down models and kinetic parameters will be able to accurately model the biophysical phenomenon of Aβ protein aggregation and identify the nucleation mass and rate constants of all the stages involved in the pathway. Our model is also reusable and will serve as the basis for making computational predictions on the system dynamics with the incorporation of other competing pathways introduced by lipids and fatty acids.

## Methods

### Reaction flux and differential equations for the reaction models

The fluxes for the set of reactions in Figure 5 can be computed as follows:

Similarly, the fluxes for the reaction set in Figure 5 is computed as:

After the kinetic schemes are established, the concentrations of various species are expressed as functions of time. The temporal change of these species can be derived from material balances and reaction kinetics. We will consider the following two models to compute the differential equations for each species in the aggregation system: (i) Simple Model: in this case, we will only be considering the reactions involved in Figure 5, i.e., the monomer addition phase. The simple model will be valid towards the beginning of the protofibril elongation phase (within the first *5 mins* or so) before the lateral association reactions take effect. (ii) Complete Model: we will consider all the reactions from Figures 5 and 6 in this case. This model is more suited to understand the dynamics of the system for a longer time-span (*30-60 mins*) for which we have the experimental measurements. Note that beyond *1 hr*, the effects of higher order oligomer addition to protofibrils as well as the detailed lateral association reactions (from Figure 7) will be more pronounced. The motivation behind the formulation of the simple model is that we can reduce the complexity of the system appreciably due to *only 3 unknown parameters:k_fb,1_, k_fb-_* and the mapping constant *b* and these estimates will be used in the complete model to estimate the remaining *2* variables: *k_la_* and *k_la-_* following our divide-and-conquer strategy.

### Differential Equations for the Simple Model

The first specie to be considered is the Aβ monomers whose rate of change is expressed in terms of its disappearance (and hence the negative sign before the summation in the corresponding differential equation) due to each of the reactions enlisted in Figure 5. The corresponding differential equation for *A_1_* depicting the rate of monomer concentration change is given by:

Similarly, the concentration change of the original protofibrils (i.e., *F* with length *~1600-mers*) is given by:

Note that the original protofibrils are only consumed in the first reaction from Figure 5. However, for each corresponding *F_i_* in the system, they are produced by the *i^th^* reaction and consumed by the *(i+1)^th^* reaction leading to the following sets of differential equations: 

Finally, the largest protofibril in the system (i.e., *F_2650_*) is only affected by the last reaction in Figure 5, and its rate of change can be expressed by:

The initial concentration of monomers is equal to the amount of Aβ added initially and along with the initial fibril concentration (i.e., concentration of *F*) is the main driving force for the downstream reactions defined in Figure 5. The concentrations of the other species are assumed to be zero at the start (i.e., *[F_i_]=0*, *i=1,…,2650*). The set of differential equations defined in the *Simple Model* contain totally *2652* variables with equal number of corresponding differential equations and initial conditions. Thus, the system of differential equations is properly defined and ready to be solved once the values of all parameters are specified. The *3* parameters that we will estimate from this model (looking into the concentration curves for the first *5 mins*) are *k_fb,1_, k_fb-_* and the mapping constant *b*.

### Differential equations for the complete model

In this case the differential equations for each of the species in the system need to consider additional reactions introduced in Figure 6. Note that, the monomers are still involved in all the reactions in Figure 5, however, none in Figure 6 involve them. Hence, the differential equation governing the rate of change of monomers remains the same as in Simple Model:

For the original protofibrils (i.e., *F*), the rate of concentration change can be calculated by taking into account all the reactions involving *F* in Figure 6 along with the first reaction from Figure 5. As a result, the time derivative of *F* equals the consumption rate due to all lateral association reactions, and the first monomer addition reaction as follows: 

The elongated protofibrils however follow a different characteristic depending on their size making it necessary to consider them separately. *F_1_,*…,*F_1050_* are involved in the *i^th^* and *(i+1)^th^* reactions in the monomer addition phase and also the *(i+1)^th^* reaction in the lateral association phase. Hence the time derivative of protofibrils with size distribution in this range equals the formation rate of the *i^th^* reaction from Figure 5 minus the consumption rate of the *(i+1)^th^* reactions from each of Figures 5 and 6 as follows:

Protofibrils in the range *F_1051_,*…,*F_1599_* however, are only affected by the reactions in Figure 5 without any contribution from the lateral association reactions. Hence the time derivative of these protofibrils equals the formation rate of the *i^th^* reaction minus the consumption rate of the *(i+1)^th^* reactions from Figure 5 as follows:

Protofibrils in the range *F_1600_,*…,*F_2649_* are again affected by the *i^th^* and *(i+1)^th^* reactions in the monomer addition phase and also the *(i-1599)^th^* reaction in the lateral association phase. Hence the time derivative of these protofibrils equals the formation rate of the *i^th^* reaction minus the consumption rate of the *(i+1)^th^* reactions from Figure 5 plus the formation rate of the *(i-1599)^th^* reaction from Figure 6 as follows:

Finally the largest protofibril (*F_2650_*) is produced by the last reactions from each of the two phases that we have considered, and its time derivative equals the production rate of the final reaction from each of Figure 5 and 6 as follows:

As before, the initial concentration of monomers is equal to the amount of Aβ added initially and that of *F* equals the initial protofibril concentration, while the concentrations of the other species are assumed to be zero to start with (i.e., *[F_i_]=0*, *i=1,…,2650*). This set of differential equations also contains *2652* variables with equal number of corresponding differential equations and initial conditions and hence is a properly defined system. However, the Complete Model is more computationally challenging to solve (due to the additional number of reactions introduced in comparison to the Simple Model) and we found it difficult to iterate through multiple runs of this model to estimate all the *5* variables (*k_fb,1_, k_fb-_, k_la_, k_la-_* and *b*). Thus it was necessary for us to define the Simple model first that will give us an estimate of *k_fb,1_, k_fb-_* and *b* as it will only require us to iterate the Complete Model lesser number of times to estimate the remaining two parameters: *k_la_* and *k_la-_*.

## Authors' contributions

PG designed the computational model for the reaction pathway and VG designed the ThT fluorescence and DLS experiments. AK helped in the experimental study and BD implemented the models in Matlab. PG and VR coordinated the study. All authors read and approved the final manuscript.

## Competing interests

The authors declare that they have no competing interests.
